# Structural investigation of borosilicate glasses containing lanthanide ions

**DOI:** 10.1038/s41598-020-64754-2

**Published:** 2020-05-12

**Authors:** M. Fabian, F. Gergely, J. Osan, T. Cendak, S. Kesari, R. Rao

**Affiliations:** 1 Centre for Energy Research, Konkoly Thege St. 29-33., Budapest, 1121 Hungary; 2 National Institute of Chemistry, Hajdrihova 19., Ljubljana, 1000 Slovenia; 3 Solid State Physics Division, Bhabha Atomic Research Centre, Mumbai, 400085 India

**Keywords:** Chemistry, Materials science

## Abstract

High level radioactive actinides are produced as a side product in reprocessing spent nuclear fuel, for which safe long-term-inert immobilizer matrices are needed. Borosilicate glasses are of great potential amongst the candidates of suitable inert materials for radioactive waste immobilization. Understanding the effects of actinide addition to a borosilicate glass matrix is of great importance in view of waste immobilization. Here we present structural studies of a simplified glass-matrix, − 55SiO_2_·10B_2_O_3_·25Na_2_O·5BaO·5ZrO_2_ - upon adding lanthanide (Ln-)oxides: CeO_2_, Nd_2_O_3_, Eu_2_O_3_, in two different concentrations 10% and 30w% each, to investigate the effects of lanthanides (Ln) taken as chemical surrogates for actinides. Neutron diffraction combined with of Reverse Monte Carlo simulations show that all investigated glass structures comprise tetrahedral SiO_4_, trigonal BO_3_ and tetrahedral BO_4_ units, forming mixed ^[4]^Si-O-^[3]^B and ^[4]^Si-O-^[4]^B linkages. ^11^B Magic Angle Spinning Nuclear Magnetic Resonance is indicative of simultaneous presence of trigonal BO_3_ and tetrahedral BO_4_ units, with spectral fractions strongly dependent on the Ln addition. Ln-addition promote the BO_3_ + O^-^→[BO_4_]^–^ isomerization resulting in lower fraction of boron in BO_3_, as compared to BO_4_ units. Raman spectra, in full agreement with neutron diffraction, confirm that the basic network structure consists of BO_3_/trigonal and SiO_4_/BO_4_ tetrahedral units. Second neighbour atomic pair correlations reveal Ce, Nd, Eu to be accommodated in both Si and B sites, supporting that the borosilicate-matrix well incorporates Ln-ions and is likely to similarly incorporate actinides, opening a way to radioactive nuclear waste immobilization of this group of elements in a borosilicate glass matrix.

## Introduction

A plethora of the useful glass properties stem from those of the network formers which constitute the bulk of the glass. These properties, however, are considerably modified by the network modifiers, which exist as single ions amongst the cross-linked network (of Si-O-B in borosilicate glasses) reducing the relative number of strong bonds leading to lower melting point, lower viscosity and modified thermal and electrical properties. This effect is of particular importance from the point of view of chemical durability of a glass.

High-level radioactive wastes (HLW) are produced by reprocessing spent nuclear fuel, which contains approximately 95% uranium, up to 1% Pu, up to 4% other actinides and various fission products. During reprocessing U and Pu are converted into a Mixed Oxide material, which can then be recycled as nuclear fuel. After reprocessing radioactive long-lived actinide elements, such as Th, U, Np, Pu, Am and Cm^1^ were identified which need treatment and long term storage in an inert host material. The borosilicate glasses, due to their mechanical and chemical durability, are widely accepted as candidates for immobilization HLW materials^2–4^. Basic and applied research is conducted world-wide to model the effects upon incorporation of such radioactive chemical constituents in their structure, determine solubility limits, study chemical durability and temperature stability of the candidate host materials.

Improved waste immobilizer borosilicate glass compositions must exhibit excellent chemical durability, higher glass transformation temperature than those currently used.

Since handling high activity radioactive actinides is not permitted in standard laboratory environments, studies are commonly performed using lanthanides (Ce^3+^, Nd^3+^ and Eu^3+^) as non-radioactive surrogates for the actinides, due to their very similar chemical properties^5,6^. Among the lanthanide ions Ce^3+^ can be used to model Pu^3+^, while Nd^3+^ and Eu^3+^ to model trivalent actinides, in particular Cm^3+^ and Am^3+^, respectively, considering their very similar ionic radii^7^. Note, that Ce is easier to reduce to its trivalent state at higher preparation temperatures, leading to both Ce^3+^ and Ce^4+^ ions^6^. In this paper, the structure of borosilicate glasses, incorporated with lanthanide oxides; CeO_2_, Nd_2_O_3_ and Eu_2_O_3_, and their effects were investigated on the structural properties of the glass network.

The 55SiO_2_·10B_2_O_3_·25Na_2_O·5BaO·5ZrO_2_ (mol%) glass matrix was earlier synthesized and its structural properties were reported^8^. In the present investigation, this glass matrix was mixed with three different lanthanide oxides i.e. CeO_2_, Nd_2_O_3_ and Eu_2_O_3_ at two concentrations (10 wt% and 30 wt%), and was characterized with the objective to understand the atomic properties and modification of the basic structure of the glass matrix upon incorporation of Ce, Nd and Eu ions. The synthesized and investigated samples are denoted as follows: *Matrix-Ce10*, *Matrix-Nd10* and *Matrix-Eu10* (for 90 wt% Matrix-glass + 10 wt% of the respective Ln oxide added), and *Matrix-Ce30*, *Matrix-Nd30* and *Matrix-Eu30* (for 70 wt% Matrix glass + 30 wt% of the respective Ln oxide added).

A variety of experimental techniques have been successfully used to determine the structure of lanthanide borosilicate glasses, among others, X-ray and neutron diffraction, X-ray fluorescence spectroscopy, EXAFS, infrared and Raman spectroscopy, nuclear magnetic resonance (NMR) spectroscopy. In the present study neutron diffraction combined with Reverse Monte Carlo (RMC) simulations, X-ray fluorescence, ^11^B Magic Spinning Nuclear Magnetic Resonance (MAS-NMR) and Raman spectroscopy were applied to determine boron, silicon and lanthanide ion-oxygen bond lengths, coordination environments, upon addition of Ce, Nd and Eu especially with the objective to model the effects of actinides on the glass matrix.

## Results and discussion

### Lanthanide concentration by XRF

The Ln elemental content of the Matrix-Ce10, Matrix-Nd10 and Matrix-Eu10 glasses obtained by XRF agreed with the nominal weighed-in concentrations within relative 5%. As an example, the measured Ce concentration for the Matrix-Ce10, was 8.02 ± 0.24 wt.%, while the nominal concentration was 8.14 wt.%, showing a deviation of less than 1.5% from the nominal composition. Other Ln-containing samples prepared with the same care are expected to have similar small deviations from the nominal composition.

### Basic glass structure with Reverse Monte Carlo simulations

All lanthanide oxide containing samples were found amorphous, no traces of crystalline phases were detected in the neutron diffraction (ND) patterns. This confirms that the simple quenching technique was sufficient. The experimental ND structure factors are compared with those derived from the RMC simulations. The overall shape of the curves within sample series (10) and (30), respectively, are fairly similar, because of the similarity in the values of the weight factors, *w*_ij_, of the partial structure factors, *S*_*ij*_(*Q*), defined below in Eqs. (1) and (2):

1 $$S(Q)=\mathop{\sum }\limits_{i,j}^{k}{w}_{ij}{S}_{ij}(Q)$$

2 $${{w}_{ij}}^{ND}=\frac{{c}_{i}{c}_{j}{b}_{i}{b}_{j}}{{[\mathop{\sum }\limits_{i,j}^{k}{c}_{i}{b}_{j}]}^{2}}$$

where *c*_*i*_*, c*_*j*,_ *b*_*i*_*, b*_*j*_ and *k* are the molar fractions, the coherent neutron scattering lengths, and the number of the elements in the sample, respectively, and *Q* is the momentum transfer. The neutron scattering length of an element is constant in the entire *Q*-range^9^. Table 1 contains the input parameters, the weighting factors for the most important atomic pairs, *w*_*ij*_ used in the RMC run for the six glass samples. The weighting factor, *w*_ij_ represents the neutron-weighted sum of the atom pairs. As seen in the table, the Si-O, B-O, Na-O and O-O atomic pairs have a large contribution to the ND intensity by which providing accurate information on the glass structure.

**Table 1:** ND weighting factors of the O-*X* and O-O partial interatomic correlations in the investigated glasses.

Samples	Weighting factor, *w*_*ij*_ (%)						
	Si-O	B-O	Na-O	Ba-O	Zr-O	Ce-O	O-O
Matrix-Ce10	16.62	9.76	13.26	1.84	2.69	2.05	40.57
Matrix-Ce30	15.22	8.86	12.07	1.66	2.38	5.62	41.51
	**Si-O**	**B-O**	**Na-O**	**Ba-O**	**Zr-O**	**Nd-O**	**O-O**
Matrix-Nd10	16.44	9.56	13.00	1.80	2.61	3.28	39.45
Matrix-Nd30	15.33	8.93	12.18	1.70	2.40	5.79	40.30
	**Si-O**	**B-O**	**Na-O**	**Ba-O**	**Zr-O**	**Eu-O**	**O-O**
Matrix-Eu10	17.01	9.92	13.54	1.86	2.67	3.57	34.03
Matrix-Eu30	14.98	8.72	11.92	1.60	2.35	9.39	32.64

Misplacements of the atoms are only accepted if they are in accordance with certain constraints (see below). The initial configuration was generated by random distribution of 10,000 atoms in a cubic simulation box. The experimentally measured number density, *ρ*_o_ was 0.065, 0.068 and 0.072 atoms Å^−3^ (corresponding to box edges of 26.79, 26.39 and 26.26 Å), for the Matrix-Ce10, Matrix-Nd10 and Matrix-Eu10 samples and 0.069, 0.071 and 0.078 atoms Å^−3^ (corresponding to box edges of 26.01, 25.89 and 25.21 Å), for the Matrix-Ce30, Matrix-Nd30 and Matrix-Eu30 samples, respectively. During the RMC runs, two types of constraints were used, the minimum inter-atomic (cut-off) distances and the Si-O tetrahedral coordination constraints. For the starting configuration, we have used the results based on our previous results for binary SiO_2_-Na_2_O^10^, B_2_O_3_-Na_2_O glasses^11^ and the Matrix glass^8^ and those of references therein. Minimum atom-atom distances (cut-off) were chosen to avoid unphysical overlapping of the atomic pairs. The coordination constrains were applied to the Si-O and B-O network former atom pairs. Based on the literature including our previous results^12–14^ it is reasonable to assume that silicon has 4-fold oxygen coordination, while coordination of B atoms by one or two O atoms were forbidden, i.e. boron atoms were assumed to be 3 or 4-fold coordinated with oxygen atoms.

**Figure 1:**
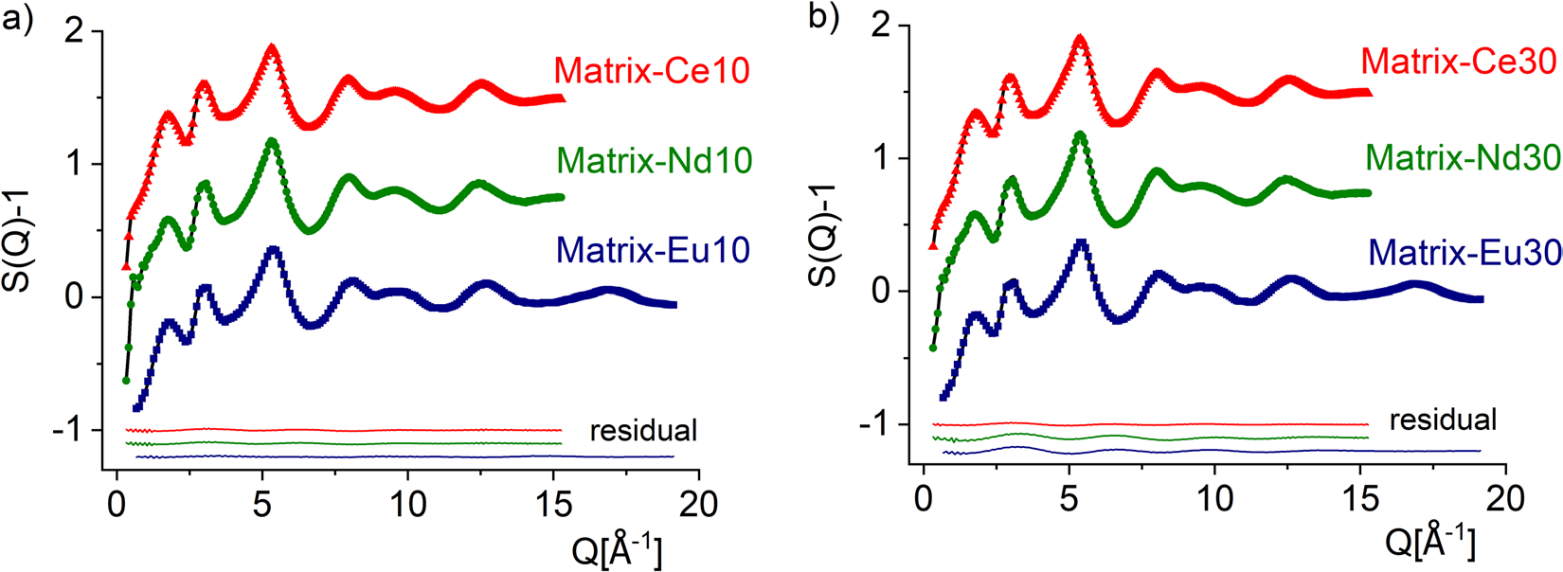
Total structure factors derived from neutron diffraction on Matrix-Ce10 (blue square), Matrix-Nd10 (green dot), Matrix-Eu10 (red triangle) (**a**) and Matrix-Ce30 (blue square), Matrix-Nd30 (green dot), Matrix-Eu30 (red triangle) (**b**) glasses (colour symbols) and RMC fits (black solid lines). For better visibility, the curves were shifted vertically.

About 30 atomic configurations were obtained from the RMC calculations of each sample corresponding to more than 1 200 000 accepted atomic displacements inside the simulation box. Figure 1 shows the ND experimental structure factor, *S*(*Q*) data for the samples along with the results of RMC simulations (Colour symbols: experimental *S*(*Q*); black line: RMC model calculation (hardly visible due to the overlap)). The convergence of the RMC calculation is very good and the final simulated *S*(*Q*) matches very well with the experimental data. The ND experimental curves are rather similar for all samples, slight differences are observed in the low-*Q* region for the intense first peak around 1.75-1.85 Å^−1^. The positions of the next peaks for all Ln-compositions scatter only in the narrow 2.95–3.00 Å^−1^ and in the 5.30–5.40 Å^−1^ ranges, respectively. The structure factor of the Ln-doped samples is of similar shape for all samples and resembles the structure factors of the SiO_2_·(B_2_O_3_)_x_·Na_2_O·BaO·ZrO_2_ glassy series presented in Ref. ^8^, where the peaks are centered at 1.95–2.00 Å^−1^, 3.00 Å^−1^ and 5.40–5.45 Å^−1^, results indicate that in sample series (10) and (30) the basic glass structures are similar with minor differences in the fine details.

**Figure 2:**
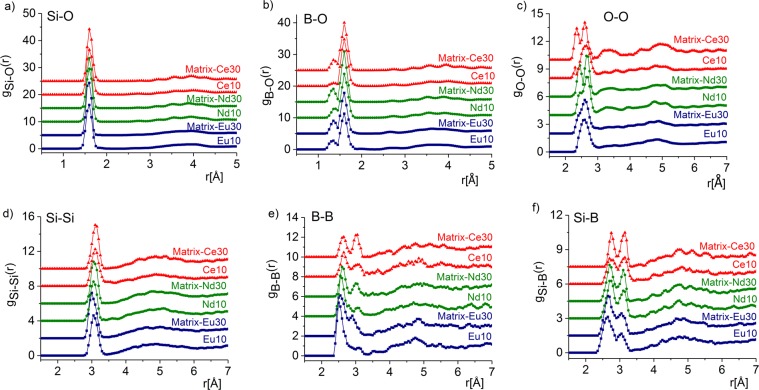
The partial atomic pair correlation functions obtained by RMC simulation for the Si-O (**a**), B-O (**b**), O-O (**c**) Si-Si (**d**), B-B (**e**) and Si-B (**f**) for the Matrix-Ce (blue square), Nd (green dot), Eu (red triangle) (10/30) glasses. For better visiblity, the curves were shifted vertically.

The overall good agreement of the RMC simulation and experimental total structure factors do not show details of the individual O-coordination of the ions. Those can be seen by investigating the partial pair distribution functions. The RMC calculation, however, provides the partial atomic pair distribution functions (for Si-O, B-O, O-O, Si-Si, B-B and Si-B correlations, *cf*. Figure (2)) and the corresponding coordination numbers.

The partial interatomic distances, the lowest-distance peak positions and the average coordination numbers derived from the nine *X*-O and the O-O partial pair correlation functions, are summarized in Table 2 and Fig. 2, respectively. The structural data for the Matrix glass sample reported earlier^8^ was re-used in the present study.

**Table 2:** Interatomic distances, *r*_ij_(Å) obtained from RMC simulations. The errors are estimated from the reproducibility of multiple RMC runs on each sample.

Samples	Interatomic distance, *r*_ij_(Å)						
	Si-O	B-O	Na-O	Ba-O	Zr-O	Ce-O	O-O
Matrix-Ce10	1.60 ± 0.01	1.35/1.59 ± 0.02	2.30/2.65 ± 0.03	2.67 ± 0.03	1.95 ± 0.02	2.20/2.67 ± 0.05	2.60 ± 0.03
Matrix-Ce30	1.61 ± 0.01	1.36/1.60 ± 0.02	2.33/2.65 ± 0.03	2.70 ± 0.03	1.96 ± 0.02	2.20/2.65 ± 0.05	2.60 ± 0.04
	**Si-O**	**B-O**	**Na-O**	**Ba-O**	**Zr-O**	**Nd-O**	**O-O**
Matrix-Nd10	1.60 ± 0.01	1.38/1.60 ± 0.02	2.30/2.65 ± 0.03	2.70 ± 0.03	1.95 ± 0.02	1.95/2.70 ± 0.05	2.40/2.65 ± 0.03
Matrix-Nd30	1.60 ± 0.01	1.35/1.60 ± 0.02	2.35/2.70 ± 0.03	2.71 ± 0.03	1.97 ± 0.02	1.95/2.70 ± 0.05	2.45/2.65 ± 0.03
	**Si-O**	**B-O**	**Na-O**	**Ba-O**	**Zr-O**	**Eu-O**	**O-O**
Matrix-Eu10	1.60 ± 0.01	1.35/1.60 ± 0.02	2.27 ± 0.02	2.65 ± 0.03	1.95 ± 0.02	2.15/2.60 ± 0.05	2.30/2.63 ± 0.03
Matrix-Eu30	1.60 ± 0.01	1.35/1.60 ± 0.02	2.29 ± 0.02	2.67 ± 0.03	1.95 ± 0.02	2.15/2.62 ± 0.05	2.35/2.60 ± 0.03

**Figure 3:**
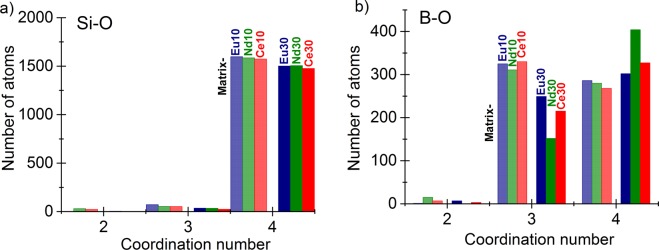
Si-O (**a**) and B-O (**b**) coordination number distributions deduced from RMC modeling for the studied glasses: Matrix-Eu (blue), Matrix-Nd (green) and Matrix-Ce (red) glasses. Light colours: 10w%, dark colours: 30w% Ln-oxides.

Si-O atomic pairs have a covalent bond length at 1.60 ± 0.01 Å in all the samples and this result is in excellent agreement with data reported in the literature^11,15,16^. For example, Si-O covalent bond length in vitreous SiO_2_ is 1.615 Å, a value somewhat shorter than that in 70SiO_2_–30Na_2_O (1.62 Å) ^11,17^ and references therein]. The average coordination number distribution shows that Si atoms have 4 oxygen atoms as nearest neighbors. The actual Si-O coordination numbers obtained from RMC modeling are 3.82, 3.84 and 3.83 (±0.02) for the Matrix-Ce10, Nd10 and Eu10, respectively and 3.94, 3.91 and 3.92 (±0.02) for the Matrix-Ce30, Nd30 and Eu30, respectively (*cf*. Figure 3a). These values confirm that the Si-O network consists of SiO_4_ units and it is very stable in these glasses. Although addition of Ln ions decreases the concentration of non-bridging oxygen slightly increasing Si-O coordination, a result consistent with the conversion of BO_3_ to BO_4_ inferred from MAS-NMR and the ND data (discussed below).

The Si-Si first neighbor distances at 3.05 ± 0.05 Å are slightly shorter than 3.10 Å found in vitreous SiO_2_ and La_2_O_3_-Na_2_O-SiO_2_ glasses^18,19^, but are in reasonable agreement with the value of 3.05 Å reported for 70SiO_2_-30Na_2_O glass^10^. The Si-O and Si-Si distances and tetrahedral coordination numbers, confirm the formation of the SiO_4_ units. The Si and B coordination numbers for all samples are summarized in Table 3.

**Table 3:** Average coordination numbers, *CN*_ij_ calculated from RMC simulation. The interval where the actual coordination number was calculated is given in brackets. The error is ~2% for Si-O and ~5% for B-O. Relative abundance (in %) were calculated from RMC configuration.

Atom pairs	Coordination number, *CN*_ij_ (atom)					
	Matrix- Ce10	Matrix-Ce30	Matrix-Nd10	Matrix-Nd30	Matrix-Eu10	Matrix-Eu30
Si-O	3.82 (*r*_1_:1.40-*r*_2_:1.80)	3.94 (*r*_1_:1.45-*r*_2_:1.80)	3.84 (*r*_1_:1.40-*r*_2_:1.80)	3.91 (*r*_1_:1.45-*r*_2_:1.80)	3.83 (*r*_1_:1.40-*r*_2_:1.82)	3.92 (*r*_1_:1.37-*r*_2_:1.80)
2-fold O- coordination	1.4	—	1.85	—	0.13	0.25
3-fold O- coordination	3.15	1.6	3.17	2.27	4.25	2.27
4-fold O- coordination	95.45	98.4	94.97	97.73	95.62	97.46
B-O	3.35 (*r*_1_:1.30-*r*_2_:1.80)	3.60 (*r*_1_:1.25-*r*_2_:1.80)	3.49 (*r*_1_:1.25-*r*_2_:1.80)	3.71 (*r*_1_:1.20-*r*_2_:1.75)	3.44 (*r*_1_:1.25-*r*_2_:1.80)	3.58 (*r*_1_:1.25-*r*_2_:1.75)
2-fold O- coordination	1.15	0.55	2.45	—	0.16	1.25
3-fold O- coordination	54.55	39.45	51.35	27.33	53.10	44.62
4-fold O- coordination	44.30	60	46.20	72.66	46.74	54.13

In spite of the complexity of the system, the B-O network was explored with good reproducibility by the RMC method including resolution of the ^3^B-O and ^4^B-O bond lengths. The B-O first neighbor distributions exhibit relatively broad nearest neighbor correlations with a characteristic peak at 1.60 ± 0.02 Å and a small shoulder at 1.35/1.40 ± 0.02 Å. The relative intensity of the two B-O peaks changes with the Ln-concentration. B-O speciation is, indeed, known to vary with changes in metal oxide concentration in borate and borosilicate glasses^12,20–22^. The nearest neighbor distance of B-B of 2.55–2.65 ± 0.02 Å is higher than 2.45 Å found in binary Na_2_O-B_2_O_3_ glasses^23^, but is in agreement with distances in MoO_3_-ZnO-B_2_O_3_ glasses (2.60 Å)^24^ and in SiO_2_-Na_2_O-B_2_O_3_ glasses (2.65 Å)^25^. A second, small emerging peak at 3.05 ± 0.05 Å of B-B correlations becomes prominent in the samples with 30 wt% of Ln oxides.

To obtain a deeper insight into the characteristic features of the B-O network, a detailed coordination number distribution analysis was performed. RMC calculations have revealed that for all glasses, the boron atoms are 3-fold coordinated (^3^B as BO_3_) and 4-fold coordinated (^4^B as BO_4_) with oxygen (Fig. 3b). The average B-O coordination numbers are 3.35, 3.49 and 3.44 (±0.05) in the Matrix-Ce10, Matrix-Nd10, Matrix-Eu10 glasses, respectively, and 3.60, 3.71 and 3.58 (±0.05) for the Matrix-Ce30, Matrix-Nd30, Matrix-Eu30 glasses, respectively (*cf*. Fig. 3b.). The analysis of coordination numbers (*cf*. Table 3) reveals useful information on the possible linkages in the glass network. For example, the relative fraction of BO_3_ and BO_4_ proves to be a sensitive probe of the basic structural units of the network. Lanthanides have been reported to act as modifiers^26,27^. The presence of ^3^B-trigonal and ^4^B-tetrahedral boron with different ratios and partial conversion of BO_3_ into BO_4_ have been reported for several borate-glasses^25–30^. A gradual change in the environment of the basic network formers was found with all the three lanthanide ions studied. The addition of lanthanides to the glass matrix promotes isomerization: BO_3_ + O^-^ → [BO_4_]^-^. The amount of O^-^ non-bridging oxygen (NBO) decreases with Ln-addition thus the network connections are modified. This kind of structural transformation establishes the ability of the glass matrix to incorporate large amount of lanthanide oxides. The highly charged state of Ln^3+^ boosts the formation of negatively charged [BO_4_]^-^ species similar to the effect of Na^+^ in sodium borate glasses^31^. The tendency of increasing relative fraction of BO_4_ at the expense of BO_3_ upon increasing the CeO_2_, Nd_2_O_3_ content was also found in our NMR studies as well (see below). Although the present multicomponent glasses are much more complicated than the usually studied binary or ternary counterparts, we succeed to consistently determine their local atomic structure.

The Si-B correlation exists at distances of ∼2.65 ± 0.05 Å and 3.15 ± 0.05 Å, which shows a possible connection between Si- and B-centered groups^15,31^. The Si-B correlation (Fig. 2f) can be interpreted as a linkage between the SiO_4_ and BO_3_/BO_4_ units constituting to the medium range order in the glass. We find that the basic trigonal BO_3_ and tetrahedral BO_4_ as well as SiO_4_, structural units are significantly correlated. Based on RMC calculations the basic network structure was established as mixed ^4^Si-O-^3^B and ^4^Si-O-^4^B linkages^8,15,25^. The network structure is stable for all studied samples build up from SiO_4_ and BO_3_/BO_4_ units.

The O-O correlations peak at two well-defined positions at 2.30 ± 0.03 Å and 2.65 ± 0.04^14,30,31^, which appear at the same O-O distances for all Ln-compositions with slight decrease of the second nearest neighbor correlation at high Ln-concentration (*cf*. Figure 3c). Neither the Na-O, nor Ba-O nor Zr-O correlations show any dependence on the Ln-concentration, (remain similar to the ones in the glass matrix) therefore those are not displayed here. A double peak exists at 2.27-2.35 ± 0.03 Å and 2.62 ± 0.03 Å in the Na-O correlation in both series of samples^8,14,32^. The Zr-O distribution peaks at 1.95 ± 0.05 Å, a distance slightly lower than 2.10 Å reported in the literature^33,34^ but it is in agreement with our previous study on the glass matrix^8^. Finally, the Ba-O distribution has a maximum at 2.70 ± 0.01 Å, a result in agreement with reported values^35–37^.

**Figure 4:**
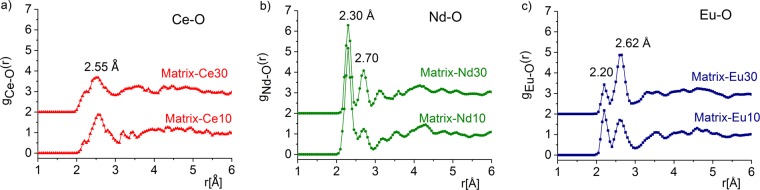
The partial atomic pair correlation functions obtained by RMC simulations for the Ce-O (**a**), Nd-O (**b**) and Eu-O (**c**) in Matrix-Ce, Nd and Eu (10/30 wt%) glasses.

The partial pair correlation distributions were also determined for the studied Ln-ions. The Ce-O, Nd-O and Eu-O pair correlation functions are illustrated in Fig. 4. The Ce-O distribution shows a broad peak at 2.55 ± 0.05 Å and 2.57 ± 0.05 Å for Matrix-Ce10 and Matrix-Ce30 samples, respectively, with a shoulder at 2.20 Å. The Ce-O inter-atomic distances vary with Ln-concentration, larger distances indicate the presence of Ce^3+^-O, in agreement with result published by another work^38^, reporting Ce^3+^-O bond length of 2.48 Å. The pre-peak at 2.20 ± 0.05 Å is specific for the Ce ions located at Si sites^39^. The average Ce coordination is 5.9 ± 0.1 and 6.1 ± 0.1 for Matrix-Ce10 and Matrix-Ce30 samples, respectively; values are close to the ones reported for Ce^3+^-O average coordination numbers to range from: 6.2 to6.5^39^.

The atomic pair correlation function of Nd-O indicates a first neighbor distance at 2.30 ± 0.02 Å, which is lower than that reported earlier^38,40^ with an additional peak at 2.70 ± 0.05 Å. The average Nd-O coordination number is 6.2 ± 0.1 and 5.6 ± 0.1 for Matrix-Nd10 and Matrix-Nd30 samples, respectively. The Nd coordination number therefore seems to decrease with increasing Nd concentration.

The Eu-O correlation function shows two peaks; one at relatively short distance, centered at 2.20 ± 0.05 Å and the second one at 2.62 ± 0.05 Å. The first peak, at 2.20 ± 0.05 Å slightly below the value of 2.22 Å on the SiO_2_-Eu_2_O_3_ system deduced from a molecular dynamics simulation^41,42^. The Eu-O coordination numbers are 5.7 ± 0.1 and 5.9 ± 0.1 for the Matrix-Eu10 and Matrix-Eu30 compositions, respectively; indicative of two short and four long bonds of the distorted octahedron.

Among the three lanthanide systems studied, the Ce-O peak is the broadest, indicating a variety of Ce-O bond lengths, whereas the Nd-O and Eu-O correlations are sharp and peak at somewhat lower distances of 2.20 Å and 2.30 Å, showings that Nd and Eu ions tend to accommodate in the glass matrix better than Ce.

It is remarkable that increasing CeO_2_, Nd_2_O_3_ and Eu_2_O_3_ addition the neutron diffraction structure factors do not show noticeable changes. The Ce-O, Nd-O and Eu-O first neighbour distances are almost identical for the two Ln-oxide added series of 10 wt% and 30 wt%. In case of Matrix-Nd (10/30) samples the first distances show more pronounced peaks than those of Matrix-Ce (10 and 30 wt%) and Matrix-Eu (10 and 30 wt%) samples. The second peak of the Nd-O and Eu-O distributions, for Matrix-Nd30 and Matrix-Eu30 samples is more pronounced as compared to the Matrix-Nd10 and Matrix-Eu10 samples, suggesting that on increasing the Ln^3+^ concentration, the Ln-O bonds get stronger at larger distances in the medium range order of the structure.

**Figure 5:**
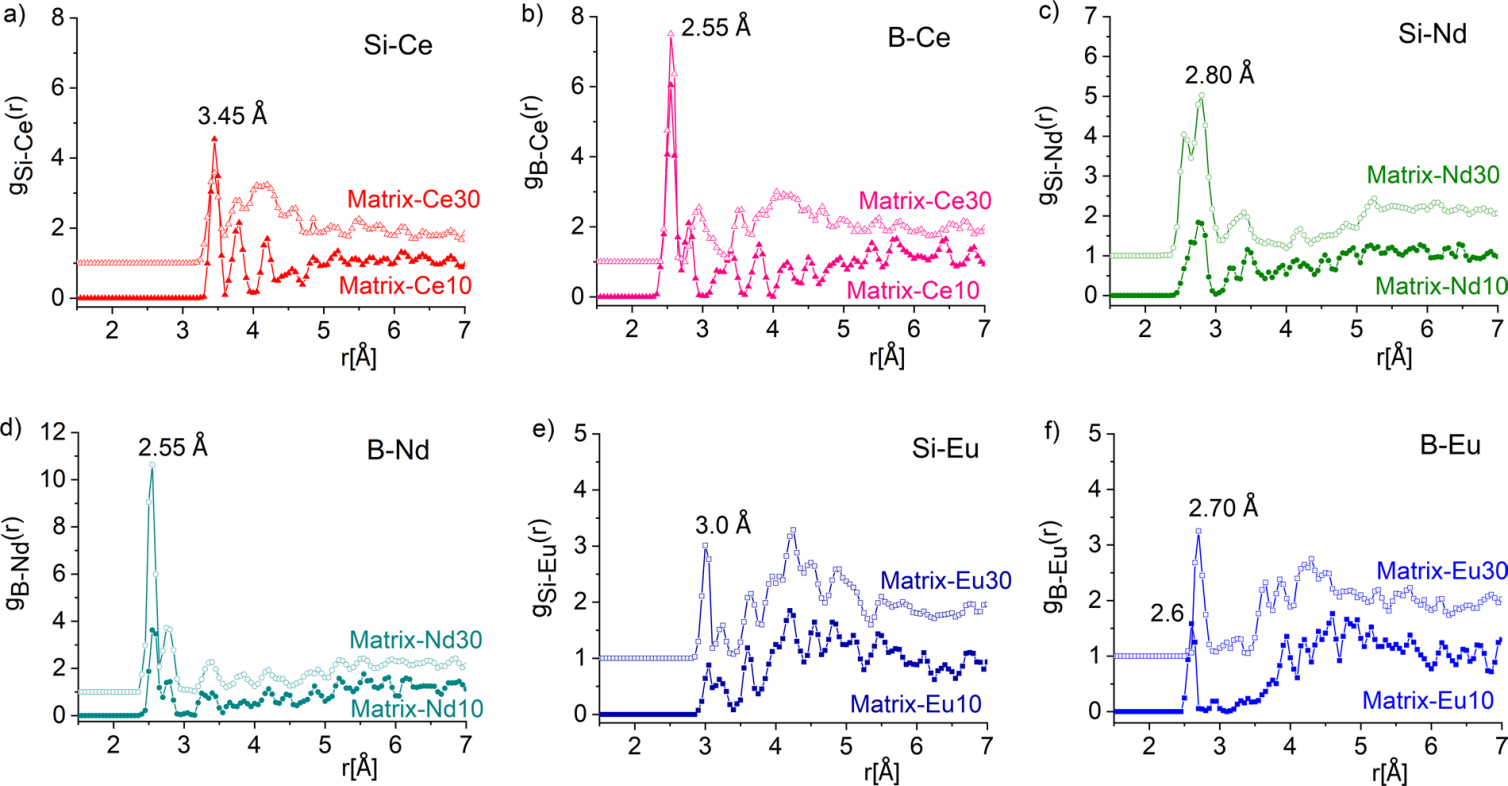
Second-neighbor distribution functions obtained by RMC simulations for the Si-Ce (**a**), B-Ce (**b**), Si-Nd (**c**), B-Nd (**d**), Si-Eu (**e**) and B-Eu (**f**) in Matrix-Ce10, Nd10, Eu10 and Matrix-Ce30, Nd30, and Eu30 glasses.

The shortest second neighbor distances are found for Si-Ce and B-Ce at 3.45 ± 0.1 Å (a value lower than 3.70 Å reported in^39^) and 2.55 ± 0.1 Å, respectively; for Si-Nd and B-Nd distances at 2.80 ± 0.1 Å and 2.55 ± 0.1 Å, respectively, and for Si-Eu and B-Eu peaks at 3.0 ± 0.1 Å and 2.60/2.70 ± 0.1 Å, respectively (presented in Fig 5 a–f). The analysis of distances shows the formation of boron-metal distances to be shorter than silicon-metal connections, suggesting that Ln-ions prefer connections to a B atom through an oxygen. Both B-Ln and Si-Ln second neighbour distances show dependence on the Ln content. Upon increasing the Ln-content the first peaks become more intense and the sub-peaks smoothen as compared to lower Ln concentration. These pronounced correlations indicate, that the Ce, Nd and Eu atoms incorporate into the glassy structure, and they are bound to oxygen at relatively short distances. The bonding arrangements and coordination environments in Matrix-Ln-oxide glasses depends on the number of terminal oxygen atoms available to connect and coordinate the modifiers, including the Ln-ions. Admittedly, these correlation functions are rather noisy due to the relatively small number of contributing atoms in the RMC simulation box. It is worth noting that these Si-Ln and B-Ln characteristic correlation function have as low as 1% weighting factor or below. However, they support Ce, Nd and Eu to incorporate the glassy structure. In conclusion, the above results accentuate the ability of the glass matrix to incorporate lanthanide ions at relatively high concentrations.

### B-O speciation from ^11^B MAS-NMR

The atoms as scattering centers contribute to the diffracted intensity in the proportion of their scattering lengths. The sharp diffraction peaks of a crystalline material allow for very accurate determination of occupation of the crystal lattice sites by a certain constituent atom of an element (of particular scattering power), however, in an amorphous material this mapping of the structure to the intensity is not as straightforward. Therefore local spectroscopic methods, like NMR may beneficially complement the diffraction data. NMR provides exclusive information on the local coordination and symmetry of and only of the probe isotope of a certain element, in the present case ^11^B spectra are exclusively characteristic of the local symmetry and coordination of boron in the Ln-doped borosilicate glasses. To understand the NMR spectra of the quadrupolar nucleus ^11^B (*I*_n_ = 3/2) the following factors have to be considered. Under MAS, the NMR spectrum is characterized by three parameters: the isotropic chemical shift δ_iso_, the quadrupolar coupling constant *C*_Q_, and the quadrupolar asymmetry parameter *η*. The two latter parameters characterize the quadrupolar interaction, which is generally of the order of several hundred kHz to several MHz for ^11^B and has therefore to be considered up to the second order. The central transition of (−1/2, +1/2) is the easiest to observe because not subjected to first-order quadrupolar interaction. Under MAS of a crystalline compound, the NMR spectrum of the central transition displays a second-order quadrupolar line shape with well-defined sharp peaks. In the case of a disordered system, however, such singularities are generally not observed because the structural disorder results in a distribution of NMR parameters, which broaden the lines being only partially narrowed by magic angle spinning. The procedure, widely applied to analyze ^11^B MAS NMR spectra of glasses, is to consider a second-order quadrupolar spectrum broadened by a Gaussian for each site, provides satisfactory results for MAS NMR.

In crystalline materials ^11^B tetrahedrally coordinated to oxygen (generally referred to as ^4^B) gives a sharp resonance in the region around δ_iso_ = 0, whereas trigonally coordinated ^3^B gives resonances in the 0–30 ppm region.

**Figure 6:**
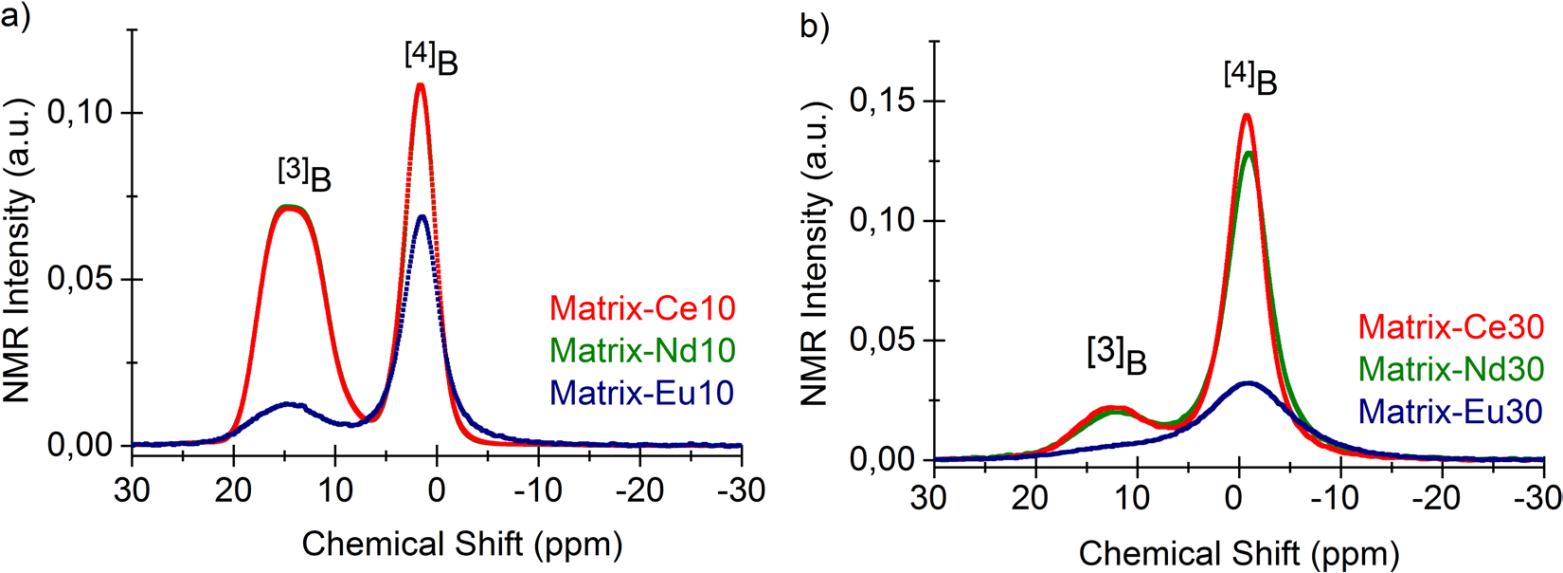
^11^B NMR spectra of Ln-doped glasses: Matrix-Ce10, Nd10 and Eu10 (**a**) and Matrix-Ce30, Nd30 and Eu30 (**b**).

Figure 6 displays ^11^B MAS-NMR spectra, for the Matrix-Ce, Nd, Eu (10 wt% and 30 wt%) samples. Two characteristic resonance peaks have been detected: a sharper line positioned around 0 ppm and a second, broader quadrupolar line between 6 and 21 ppm consistent with ^4^B and ^3^B oxygen coordination, respectively. ^11^B MAS-NMR spectra for all the glass samples, therefore – in agreement with the literature^15,16^ – are indicative of simultaneous presence of trigonally coordinated ^3^B and tetrahedrally coordinated ^4^B boron in (BO_3_) and (BO_4_) structural units. The peak intensities show strong concentration dependence for all Ce^3+^, Nd^3+^ and Eu^3+^ lanthanide ions. The ^3^B peak strongly decreases while the ^4^B peak strongly increases with increasing Ce, Nd, and Eu concentrations of the matrix glass.

**Figure 7:**
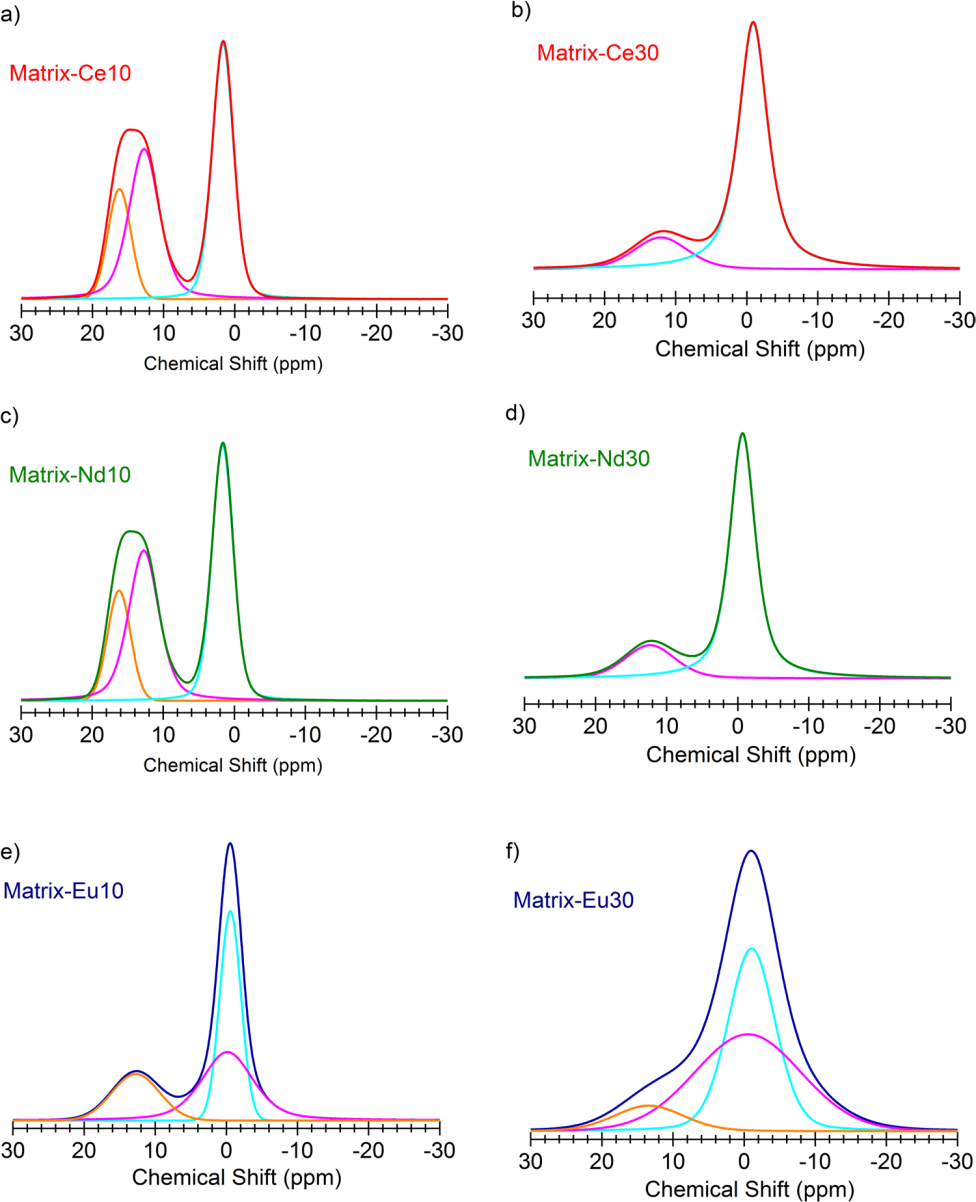
Deconvoluted and peak fitted ^11^B MAS-NMR spectra of glasses. Simple symmetric Gaussian broadening assumed.

In the 30w% europium sample, both peaks are considerably broadened, therefore the ^3^B and ^4^B contributions can only be determined with large uncertainties. This is certainly due to the fact that Eu^3+^ ions are magnetic and the interaction between the nuclear and the ionic moments broaden the spectrum when the Eu atoms are sufficiently close to each other in the Eu30 sample. The NMR spectra for all the samples were de-convoluted assuming symmetric Gaussian broadening (*cf*. Figure 7). The integrated area under these peaks was taken as 100% to calculate the spectral fractions and the coordination number of B in the samples^43^.

The effect of the cation to boron speciation can be quantified by the spectral fraction of ^3^B to ^4^B, and the corresponding coordination number (see Table 4). One can observe that with increasing Ln concentration the ^3^B/^4^B spectral fraction decreases and the B-O coordination number increases in all samples. In case of Ce and Nd samples, the conversion of BO_3_ trigonal units into BO_4_ tetrahedral units is significant, while – with a large error bar, due to the overlap – one may speculate that the Eu_2_O_3_ addition to the Matrix glass results in smaller specific conversion of ^3^B into ^4^B units.

**Table 4:** B-O coordination numbers, *N*_B-O_ derived from ^11^B NMR spectral fractions of ^3^B and ^4^B.

	Coordination number, *N*_B-O_					
	Matrix-Ce10	Matrix-Ce30	Matrix-Nd10	Matrix-Nd30	Matrix-Eu10	Matrix-Eu30
^3^B/^4^B	56.7/43.3	17.1/82.9	56.9/43.1	18.8/81.2	31.5/68.5	25.0/75.0
N_B-O_	3.43	3.83	3.43	3.81	3.68	3.75

^11^B MAS-NMR results indicate a strong dependence on the concentration of all investigated CeO_2_, Nd_2_O_3_ and Eu_2_O_3_ oxides in the borosilicate Matrix glass, thus Ln ions, in full accordance with the RMC modeling of the neutron diffraction data, strongly effect the boron environment, and, consequently, the network structure of the borosilicate glass.

### Raman spectroscopic analysis

**Figure 8:**
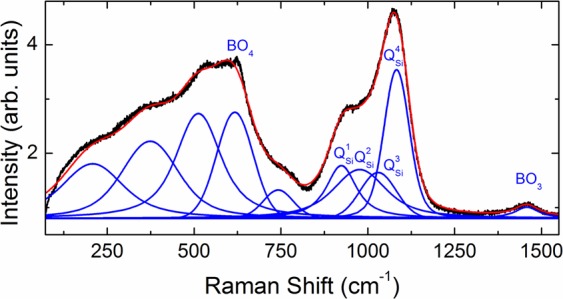
Raman spectrum of the borosilicate glass matrix recorded at ambient temperature. The Voigt functions corresponding to the individual modes are marked in blue and the envelope fit curve in red.

Information on the dynamics of network structure of glasses can be obtained from vibrational spectra. Raman spectroscopy is ideally suited to delineate the glass network especially in the high-frequency regime (850–1200 cm^−1^) corresponding to Si-O stretching modes of *Q*^n^ species. Introduced by Ref. ^44^ *Q*^n^ (*n* = 0, 1, 2, 3 or 4) is the Si-O species in tetrahedral SiO_4_ units with *n* bridging oxygen atoms per silicon. The Raman spectrum of sodium borosilicate glasses contains broad features in the three regions, 350–800, 850–1200 and 1300–1650 cm^–1^ corresponding to O-Si-O bending (around 500 cm^−1^) as well as to BO_4_, *Q*^n^ and BO_3_ wave number regions, respectively. Figure 8 shows the phonon-population-corrected Raman spectrum of the glass matrix recorded at ambient temperature. The spectrum was de-convoluted using eleven Voigt functions corresponding to the individual vibration modes labeled BO_4_, *Q*^1 to 4^ and BO_3_, according to^44^. These wave numbers agree well with those reported in earlier studies^44–48^. In a polarized Raman spectroscopic study of danburite^45^, a mineral of Ca_2_B_2_Si_2_O_8_ composition, the strong band in sodium borosilicate glasses around 630 cm^−1^ was assigned to the breathing mode of the danburite-like rings consisting of two SiO_4_ and two BO_4_ tetrahedral, charge compensated by sodium rather than calcium^46^. The hump at 770 cm^−1^ was attributed to four-coordinated boron in six-member diborate and boroxol rings^46^. This assignment is based on studies of the effects of Na^+^, addition, which resulted in an increased intensity of the 770 cm^−1^ band interpreted as due to the formation of BO_4_^-^ at the expense of BO_3_ units^46,48^.

**Figure 9:**
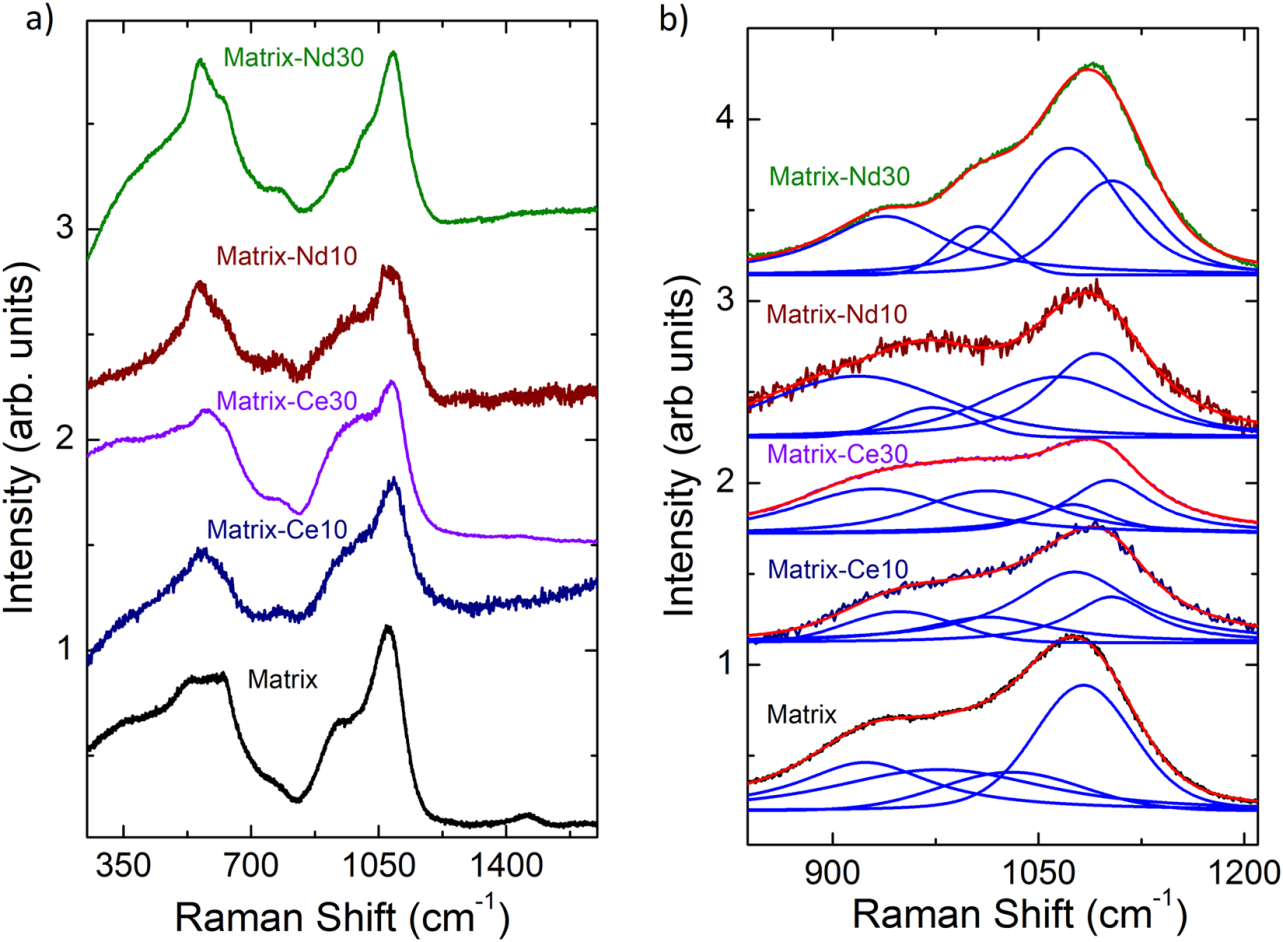
Raman spectra of glass matrix along with Matrix-Ce10, Nd10 and Matrix-Ce30, Nd30 (**a**) and their respective fitting with Voigt functions in the Q^n^-region (**b**).

Lanthanides are known to act as network modifiers in the glass matrix^49^. Raman spectra of the glass matrix as well as of the studied lanthanide-containing glasses (Matrix-Ce10, Matrix-Ce30, Matrix-Nd10 and Matrix-Nd30) are shown in Fig. 9. The Ln-glasses were observed to be homogeneous and optically clear and did not show any visible clustering of CeO_2_ or Nd_2_O_3_ precipitates. There are only minor changes in the band positions indicative of minor changes in the bond lengths in the polyhedra upon changing from one Ln ion to the other, in accordance with the ND and NMR results. However, the relative intensities of the bands at 350–800, 850–1200 and 1300–1650 cm^–1^ change with lanthanide concentration due to changes in B-O and Si-O speciation. The extremely broad lines in the Raman spectra of Matrix-E10 and Matrix-Eu30 glasses prevented an unambiguous identification of transitions. Therefore those spectra are not presented here. Instead, an independent photoluminescent spectroscopic investigation was performed on the Eu-glasses, which are presented after the Raman results. It can be seen in Fig. 9a that the positions of bands in the *Q*^n^ region in the glass matrix shift to slightly higher frequencies in glasses doped with Nd_2_O_3_ and CeO_2_ but major structural changes were not observed in the network. Since the spectra were recorded with two different wavelengths of the light source for the Nd and Ce glasses, we have compared the ratios of deconvoluted *Q*^n^ species within the spectra. In Fig. 9b the relative intensity of the first three modes are seen to increase with respect to *Q*^4^ and as a function of Ln-doping. This is in agreement with an earlier study on La-doped sodium borosilicate glasses^50^ in which the intensity was found to increase for lower frequency bands at the expense of the high frequency band in the *Q*^n^ region interpreted as due to incipient de-polymerization.

**Figure 10:**
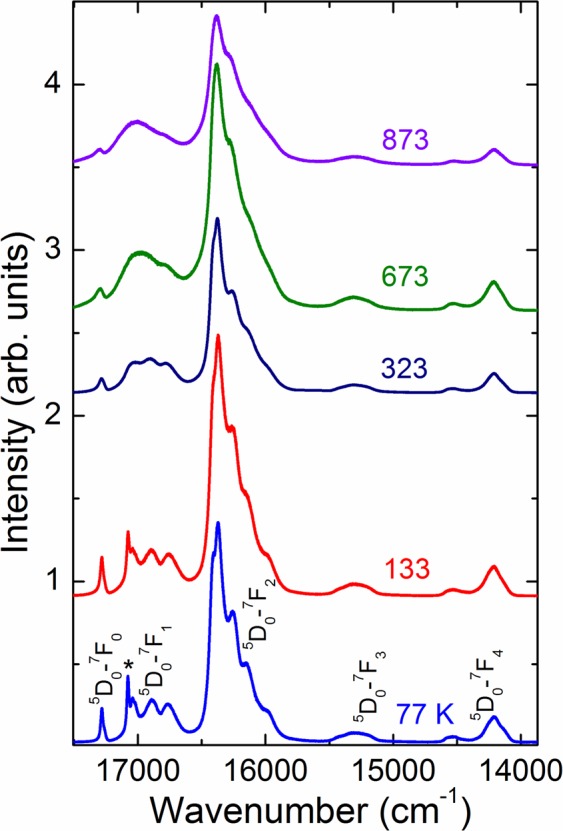
Europium luminescence spectra of the Matrix-Eu30 borosilicate glass at temperatures between 77 to 873 K. The peak marked with asterisk (*) at 17076 cm^−1^ at 77 K is assigned to emission from higher excitation levels of Eu^3+^^51^.

Eu has been reported to be extremely sensitive for minor structural changes. Thus, Eu^3+^ is an excellent ion for luminescence studies in glasses because of its unique level structure with non-degenerate ground (^7^F_0_) and excited (^5^D_0_) states and being very sensitive to the local symmetry of the Eu^3+^ ion^51^. As compared to a crystalline lattice, in general, the Eu^3+^ luminescence lines in Eu-doped glass are broad. In a crystalline compound, Eu^3+^ luminescence spectra have been used to detect and phase transitions and small distortions in the crystal structure. Temperature dependence of Eu^3+^ spectra may also be informative of the mechanism of the broadening of the spectral lines. By lowering the temperature the overlaps due to thermal vibrations are lifted, resulting in narrow luminescence lines. Figure 10 shows europium luminescence spectra of the Matrix-Eu30 glass. In order to unambiguously assign the luminescence lines, spectra were also recorded at cryogenic temperatures. Five peaks were observed and assigned to 4 *f* transitions of Eu^3+^ ions, namely ^5^D_0_ → ^7^F_*J*_ (*J* = 0, 1, 2, 3, 4)^51^ in the temperature range of 77 K to 873 K, as shown in Fig. 10. In the spectra collected at 77 K, all luminescence peaks are well resolved to Voight functions. The presence of a single line in the ^5^D_0_ → ^7^F_0_ region and three lines in ^5^D_0_ → ^7^F_1_ region reveal that there exist only one site available for Eu^3+^ in the glass matrix. The ^5^D_0_ → ^7^F_1_ splitting is about 400 cm^−1^ a typical value expected for a glass. One additional peak appearing at 77 K may be the one assigned in the literature^51^ to be due to emission from higher excitation levels of Eu^3+^ (*cf*. peak marked with asterisk (*) in Fig. 10). The ^5^D_0_ → ^7^F_2_ peak dominates the spectra in the entire temperature range. Its intensity is “hypersensitive”^51^ to changes of the local symmetry and to the nature of the ligands as compared to other electric-dipole transitions, while the intensity of the ^5^D_0_ → ^7^F_1_ band of magnetic-dipole origin is independent of the local symmetry. The absence of emission for higher ^5^D_J_ levels can be related to multiphonon or cross-relaxation processes, caused by a relatively high concentration of Eu^3+^ centers in the glass network. From the number of distinct peaks in the luminescence spectra, it appears that the europium site is of C_1_, C_s_ or C_2_ point symmetry^51^. The summed peak intensity ratio of the ^5^D_0_ → ^7^F_2_ and ^5^D_0_ → ^7^F_1_ regions is more than 2 in the entire temperature range which is an indication of low symmetry of the Eu^3+^ site^52,53^.

With increasing temperatures, the full width at half maximum (FWHM) of the ^5^D_0_ → ^7^F_0_ transition considerably increases from 26 cm^−1^ at 77 K to 50 cm^−1^ at 298 K, while inhomogeneous broadening is known to cause, minor increase in FWHM of ^5^D_0_ → ^7^F_0_ peak with the increase of temperature^54^. Europium luminescence spectra of Matrix-Eu10 and Matrix-Eu30 glasses are of similar shape indicating similar local environment of europium independent of the Eu concentration.

## Conclusions

Borosilicate matrix glass (55SiO_2_·10B_2_O_3_·25Na_2_O·5BaO·5ZrO) with 10 wt% and 30 wt% addition of lanthanide oxides of CeO_2_, Nd_2_O_3_ and Eu_2_O_3_, respectively were prepared, characterized by density measurements and X-ray fluorescence and their structural analysis was carried out by neutron diffraction, ^11^B MAS-NMR, Raman and photoluminescence spectroscopy. The investigations reveal that the borosilicate network has the ability to incorporate a large number of Ln ions. The incorporation is accompanied by a considerable modification of the borate network via the BO_3_ + O^-^→[BO_4_]^–^ isomerization reaction. Maximum conversion of BO_3_ into BO_4_ units is produced by Ce ions, a result which is found by both neutron diffraction and ^11^B NMR studies.

The RMC simulation in accordance with experimental neutron diffraction data show the basic network structure of the Ln-oxide-doped borosilicate glasses to consists of mixed ^3^B-O-^4^Si and ^4^B-O-^4^Si chain segments, but the fractions of BO_3_ and BO_4_ units strongly depend on the Ln-oxide content in such a way that formation of BO_4_ is enhanced upon increasing the Ln-oxide content. The derived Ce-O, Nd-O and Eu-O bond distances and coordination numbers as well as the temperature dependent Eu^3+^ luminescence studies reveal that only one site is available for Eu^3+^ in the glass matrix network. Moreover, the second nearest neighbor atomic pair correlations established between cerium, neodymium, europium and the network forming (Si, B) atoms accentuate that the Ln-doped glasses exhibit a stable basic network structure. Therefore one may conclude that the investigated borosilicate glass matrix may incorporates large concentration of actinides thus it has a great potential to be used in high level nuclear waste management.

## Methods

### Sample preparation

**Figure 11:**
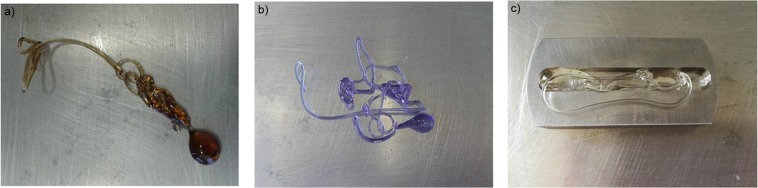
Poured glasses for the Matrix-Ce30 (**a**), Matrix-Nd30 (**b**) and Matrix-Eu30 (**c**) compositions.

Since actinides are radioactive and not easily available, lanthanides (Ln) ions were used in this study to model the effects on the structure of the model borosilicate glass matrix. Ln ions have similar atomic radii and masses to their actinide counterparts, therefore they are good surrogates of the actinides. The raw materials used to prepare the samples were of analytical grade, SiO_2_, Na_2_O, BaO, ZrO_2_, B_2_O_3_, CeO_2_, Nd_2_O_3_, Eu_2_O_3_ were purchased from Sigma-Aldrich Company. B_2_O_3_ was 99.6% isotope enriched in ^11^B^55^. The batch mixtures were melted in platinum crucible in a suitable electrical furnace at 1300 to 1450 °C. Bulk, highly transparent, colorful glass samples were prepared by melt-quenching. About 8–10 g of the melt was poured on a stainless steel plate and let it solidify. Figure 11 shows a set of just solidified samples. The bulk samples were comminuted by ball-milling (Retsch MM400), using agate balls to a particle size below 50 µm. Two series of samples were synthesized and investigated:

Series(10): 90 wt%[Matrix] + 10 wt%CeO_2_, 90 wt%[Matrix] + 10 wt%Nd_2_O_3_ and 90 wt%[Matrix] + 10 wt%Eu_2_O_3_, where the matrix composition was 55SiO_2_·10B_2_O_3_·25Na_2_O·5BaO·5ZrO_2_ (mol%), (referred to as Matrix-Ce10, Matrix-Nd10, Matrix-Eu10 samples, respectively), and

Series(30): 70 wt%[Matrix] + 30 wt%CeO_2_, 70 wt%[Matrix] + 30 wt%Nd_2_O_3_ and 70 wt%[Matrix] + 30 wt%Eu_2_O_3_ (referred to as Matrix-Ce30, Matrix-Nd30, Matrix-Eu30 samples, respectively).

The density of the glasses was determined gravimetrically at 22±0.5 °C using an electronic balance of 10^–4^ g sensitivity with distilled water as immersion fluid. Density measurement of each sample was performed four times, resulting is a standard deviation below 0.01 g·cm^−3^. The atomic number density used in the RMC analysis was calculated from the mass densities. The structural properties of the borosilicate glass matrix were reported earlier^8^.

### X-ray fluorescence (XRF) analysis

XRF analysis was carried out on pressed pellets using an in-house system based on a standard Mo-anode X-ray diffraction tube (Seifert) with Mo secondary target and with Cartesian Geometry optics. Fluorescent X-ray photons were collected using a silicon drift detector (KETEK, Munich, Germany) with an energy resolution of 150 eV for Mn-K_α_ X-rays^56^. Pellets of 20 mm diameter were pressed from 0.5 g of Matrix-Ce10, Matrix-Nd10 and Matrix-Eu10 samples adding 0.5 g boric acid as binder and diluting agent to each. X-ray fluorescence spectra were acquired for 5000 s. Standard foils containing known amounts of single elements or simple compounds (Micromatter, Canada) were measured to obtain the elemental sensitivity curves. Matrix absorption corrections were performed for calculation of the elemental concentrations. Due to the granularity of the pellets, concentrations of heavier elements are underestimated due to the grain size effect, requiring additional corrections. Since the analyte line positions are close to each other (Ba-Lα 4.46 keV, Ce-Lα 4.84 keV, Nd-Lα 5.23 keV and Eu Lα 5.85 eV), and the Ba content of the matrix glass is known, Ba concentrations were accepted as nominal and the Ln-concentrations were calculated relative to Ba. The overall uncertainty of the method was 3% relative.

### Neutron diffraction experiments

Neutron diffraction (ND) measurements were performed on the PSD diffractometer (λ_0_ = 1.068 Å)^57^ at the Budapest Neutron Centre, and the 7C2 diffractometer at the Laboratoire Léon Brillouin, Saclay (λ_0_ = 0.726 Å)^58^. The powder specimens of about 3–4 g each were filled in thin-walled cylindrical vanadium sample holders of 8 mm and 6 mm diameter, respectively. The raw data were corrected for detector efficiency, background scattering and absorption effects. The total structure factor, *S(Q)* was calculated by local software packages. Detailed data evaluation was presented earlier^8^.

### Reverse Monte Carlo simulations

The Reverse Monte Carlo (RMC) simulation is a powerful technique to build large 3D structural models in accordance with experimental data, in particular total structure factors (*S*(*Q*)) obtained from diffraction experiments^59^. The *S(Q)* data were simulated for neutron diffraction by the RMC^++^ code^60^. The RMC algorithm calculates the *g*_ij_(*r*) one-dimensional partial atomic pair correlation functions, and by inverse Fourier transformation, calculates the *S*_ij_(*Q*) partial structure factors as:

3 $${S}_{ij}(Q)=1+\frac{4\pi {\rho }_{0}}{Q}{\int }_{0}^{{r}_{\max }}r\,[{g}_{ij}(r)-1]\,\sin \,Qr\,dr$$

where *ρ*_0_ and *r*_max_ are the atomic number density and the half-edge-length of the simulation box in the RMC calculation. The actual computer configuration is modified by moving the atoms randomly until the calculated *S*(*Q*) and experimental data agree within the experimental error (*cf*. Equations (1) and (2)).

### MAS -NMR spectroscopy

NMR spectroscopy is an ideal tool to study the short range structure of glasses, since the ^29^Si, ^11^B and ^17^O are all sensitive NMR probes. ^11^B NMR spectroscopy has long been used in relation to study geometrical and substitutional disorder of and around B, which – in the form of B_2_O_3_ – is a network forming component in borosilicate glasses. The NMR chemical shift is sensitive to the probe atom’s local environment; however, much detail is lost in the conventional NMR technique due to various mechanisms which broaden the resonances from different environments. The ‘magic angle spinning’ (MAS) technique eliminates the dipolar contribution to broadening and also reduces the effect of chemical shift anisotropy and quadrupole broadening.

^11^B MAS-NMR studies of the investigated lanthanum-oxide-doped borosilicate glasses were carried out with 600 MHz Varian NMR System (192.5 MHz for ^11^B) equipped with the 3.2 mm Double Resonance MAS probe installed at the Slovenian NMR Centre in Ljubljana, Slovenia^61^. Spectra were acquired with single pulse sequence using a short non-selective 0.6 μs pulse and XiX decoupling during acquisition to observe the central (−1/2, +1/2) transition of ^11^B (*I*n = 3/2). The sample rotation frequency was 20 kHz and the relaxation delay was chosen to be 10 s. 200 scans were accumulated in each spectra.

Chemical shift are of positive values downfield, relative to that of an external sample of boric acid solution.

### Raman and luminescence spectroscopy

A high-throughput micro-Raman spectrometer (JY-Model LabRam HR-800 Evolution) was used to record the Raman spectra in the backscattering geometry using 532/633 nm excitation source. A charge-coupled device was used to detect the scattered light in the Raman shift range of 50–1600 cm^**−**1^. A 50× microscope objective lens was used to focus the laser beam onto the sample. Raman and luminescence spectroscopy measurements were carried out on powder samples of the glasses. Light power on the sample was about 10 mW for all Raman spectra. In order to account for the finite temperatures of the measurements, the intensity of the observed Stokes Raman spectra acquired at temperature *T* were divided with the [*n*(*ω*) + 1] Bose-Einstein population factor^62^ where *n*(*ω*) = [exp(*ħω*/*kT*) − 1]^−1^. The reduced Raman spectra were fitted to Voigt line shapes to determine the position, line width and intensity corresponding to the various vibrational modes. Raman spectra of the Nd compounds were recorded using 633 nm laser to avoid strong luminescence lines. For the Eu^3+^ luminescence measurements, the sample was placed in a Linkam THMS600 temperature-controlled heating/cooling stage for carrying out experiments ranging from 77 to 873 K.
